# MRI-Derived Pancreatic Fat Fraction Is Independently Associated with Intraductal Papillary Mucinous Neoplasms: A Case–Control Study

**DOI:** 10.3390/diagnostics16142198

**Published:** 2026-07-14

**Authors:** Sedat Çiçek, Delyadil Karakaş Kılıç, Selman Çetin, Abdulvahap Hohluoğlu, Furkan Kırsoy, Jehat Kılıç, Abdullah Mubin Özercan, Mustafa Yıldırım, Mehmet Yalnız, İbrahim Halil Bahçecioğlu

**Affiliations:** 1 Department of Gastroenterology, Fırat University, Elazig 23119, Türkiye; drsedatcicek23@gmail.com (S.Ç.); drselmancetn@gmail.com (S.Ç.); vahap.hohluoglu@gmail.com (A.H.); f.kirsoy@hotmail.com (F.K.); mubinozercan@gmail.com (A.M.Ö.); mehmetyalniz@hotmail.com (M.Y.); ihbahcecioglu@yahoo.com (İ.H.B.); 2 Department of Oncology, Dicle University, Diyarbakir 21280, Türkiye; karakasdelyadil@gmail.com; 3 Department of Rheumatology, Fırat University, Elazig 23119, Türkiye; 4 Department of Radiology, Fırat University, Elazig 23119, Türkiye; mustafa23468@outlook.com

**Keywords:** intraductal papillary mucinous neoplasms, pancreatic steatosis, magnetic resonance imaging, biomarkers

## Abstract

**Introduction:** Intraductal papillary mucinous neoplasms (IPMNs) are precursor lesions of pancreatic cancer. This study investigated the association between MRI-derived pancreatic fat fraction and IPMN. **Materials and Methods:** This retrospective case–control study included 60 patients with IPMN and 120 controls evaluated between 2018 and 2025. All participants underwent pancreatic MRI with proton density fat fraction (PDFF) imaging. Pancreatic fat fraction was measured in the pancreatic head, body, and tail on PDFF maps, and mean pancreatic fat fraction was calculated. Group comparisons, receiver operating characteristic analysis, and logistic regression analyses were performed to evaluate the association between pancreatic fat fraction and IPMN. **Results:** A total of 180 participants were included in the study, comprising 60 patients with IPMN and 120 controls without IPMN. Patients with IPMN were significantly older than controls (72.5 vs. 57.0 years, *p* = 0.001). The IPMN group demonstrated higher glucose and LDH levels and lower HDL cholesterol, hemoglobin, hematocrit, and platelet counts compared with controls (all *p* < 0.05). Pancreatic fat fraction measurements were significantly increased in patients with IPMN across all pancreatic regions. Head, body, tail, and mean pancreatic fat fractions were markedly higher in the IPMN group than in controls (all *p* = 0.001). In multivariable logistic regression analyses, age and pancreatic fat fraction remained independently associated with IPMN presence. ROC analysis demonstrated excellent diagnostic performance, with mean pancreatic fat fraction showing the highest discriminative ability (AUC = 0.968), followed by head (AUC = 0.934), body (AUC = 0.922), and tail fat fraction (AUC = 0.876). **Conclusions:** Pancreatic fat fraction was significantly higher in patients with IPMN and remained independently associated with IPMN presence after multivariable adjustment. Mean pancreatic fat fraction demonstrated excellent diagnostic performance for distinguishing IPMN from non-IPMN individuals. Quantitative MRI-based assessment of pancreatic fat may represent a useful imaging biomarker for IPMN detection.

## 1. Introduction

Pancreatic ductal adenocarcinoma (PDAC) remains one of the deadliest malignancies worldwide because of its poor prognosis and late presentation. Since most PDACs arise from identifiable precursor lesions, improving the detection and characterization of these lesions has become a major focus of current research [1]. Therefore, the identification of imaging biomarkers and risk factors that may facilitate the early detection of pancreatic cancer has become a major focus of current research.

Pancreatic cancer is widely recognized to arise predominantly from precursor neoplastic lesions. Among these, pancreatic intraepithelial neoplasia (PanIN) and intraductal papillary mucinous neoplasms (IPMNs) are considered the most important precursor lesions [2]. In particular, IPMNs represent an important model for understanding pancreatic carcinogenesis because they can be detected using imaging modalities and possess the potential for malignant transformation. Although several clinical and radiological criteria are currently available for assessing malignancy risk in IPMNs, their diagnostic performance remains suboptimal, and there is an ongoing need for novel biomarkers to improve the identification of high-risk lesions and advanced neoplasia [3].

Pancreatic steatosis is characterized by abnormal fat accumulation within the pancreatic parenchyma and is closely associated with metabolic disorders such as obesity, metabolic syndrome, and type 2 diabetes mellitus. In recent years, pancreatic fat accumulation has been recognized not only as a manifestation of metabolic dysfunction but also as a condition that may contribute to chronic inflammation, cellular injury, and neoplastic transformation [4]. Accumulating evidence suggests that pancreatic steatosis may be a potential risk factor for pancreatic neoplasia, particularly PDAC [4].

Magnetic resonance imaging (MRI)-based proton density fat fraction (PDFF) measurements enable the noninvasive and quantitative assessment of pancreatic fat content and have been shown to correlate strongly with histopathological findings, making them a reliable imaging modality [5]. Recent studies have demonstrated that MRI-derived pancreatic fat fraction is independently associated with the presence of pancreatic precursor lesions. These findings suggest that pancreatic fat accumulation may serve as a novel imaging biomarker associated with pancreatic neoplastic development [5].

Despite these findings, the relationship between pancreatic steatosis and IPMNs, as well as the underlying biological mechanisms, remains incompletely understood. Clarifying the role of pancreatic fat accumulation in the development of IPMNs may contribute to the identification of individuals at increased risk for pancreatic cancer and facilitate the development of early detection strategies. Therefore, this study aimed to investigate the association between pancreatic steatosis and IPMNs and to evaluate the potential utility of pancreatic fat fraction as an imaging biomarker for pancreatic neoplasia.

## 2. Materials and Methods

This retrospective case–control study included a total of 180 patients who were evaluated between January 2018 and December 2025. The study population consisted of 60 patients diagnosed with intraductal papillary mucinous neoplasm (IPMN) and 120 control subjects without IPMN. Clinical, metabolic, laboratory, and radiological data were retrospectively collected and compared between the IPMN and non-IPMN groups. The study was conducted in accordance with the principles of the Declaration of Helsinki and was approved by the local ethics committee on 25 December 2025 with decision number 19–20.

Demographic data (age and sex), anthropometric measurements (body mass index and waist circumference), and biochemical and hematological parameters, including glucose, total cholesterol, HDL cholesterol, LDL cholesterol, triglycerides, urea, creatinine, AST, ALT, ALP, GGT, LDH, amylase, lipase, white blood cell count, hemoglobin, hematocrit, and platelet count, were retrospectively extracted from the hospital’s electronic health record system (ENLIL; Republic of Türkiye Ministry of Health, Ankara, Türkiye). Clinical, laboratory, and radiological data were reviewed and recorded for all eligible participants using standardized data collection procedures.

Inclusion Criteria:

- Presence of a cystic lesion associated with the pancreatic ductal system on MRCP
- Diagnosis confirmed by at least 12 months of radiological follow-up
- Availability of a proton density fat fraction (PDFF) sequence in the same MRI examination
- Age between 18 and 75 years
- Body mass index (BMI) between 19 and 25 kg/m^2^.

Exclusion Criteria:

- History of acute pancreatitis within the previous 6 months
- History of pancreatic resection
- Presence of a space-occupying lesion in the pancreas
- Diffuse atrophic pancreatitis
- Significant imaging artifacts affecting image quality
- Conditions markedly affecting systemic fat metabolism, such as advanced malignancy or cachexia
- Presence of metabolic syndrome
- History of smoking or excessive alcohol consumption.

IPMN Group: Patients who underwent pancreatic magnetic resonance imaging (MRI) between January 2018 and December 2025 and were diagnosed with main duct intraductal papillary mucinous neoplasms (MD-IPMNs) based on radiological findings were included. Only patients with MD-IPMNs were enrolled because this subtype carries a substantially higher risk of malignant transformation than branch duct IPMNs and is more reliably diagnosed using current imaging criteria, thereby providing a more homogeneous study population and reducing potential diagnostic misclassification. The diagnosis of IPMN was established based on MRI findings and radiological reports reviewed by experienced radiologists.

Control Group: The control group consisted of individuals who underwent pancreatic MRI during the same study period and were reported to have a radiologically normal pancreas, without evidence of pancreatic cystic lesions, IPMN, or other known pancreatic diseases. These individuals were selected from the same patient population and served as controls for comparison with the IPMN group.

MRI Acquisition Protocol

All magnetic resonance imaging (MRI) examinations were performed using 3.0-Tesla MRI (Philips Healthcare, Best, the Netherlands) systems according to a standardized imaging protocol. The protocol included the following sequences:

- Axial T1-weighted images
- Axial and/or coronal T2-weighted images
- Magnetic resonance cholangiopancreatography (MRCP) sequences
- Multi-echo gradient-echo Dixon sequences for quantitative fat assessment.

Pancreatic fat content was quantitatively measured using proton density fat fraction (PDFF) maps generated from the Dixon sequences. All measurements were obtained from dedicated PDFF maps to ensure objective and reproducible assessment of pancreatic fat deposition.

Pancreatic Fat Fraction Measurement

MRI examinations were performed using a standardized pancreatic MRI protocol on a 3.0-T scanner (Philips Healthcare, Best, the Netherlands). PDFF maps were generated from a multi-echo Dixon sequence according to the manufacturer’s reconstruction algorithm. Pancreatic fat fraction measurements were independently performed by two experienced abdominal radiologists who were blinded to the clinical characteristics and diagnostic status of the participants. The diagnosis and imaging evaluation of intraductal papillary mucinous neoplasms (IPMNs), including assessment of main pancreatic duct diameter and associated imaging features, were performed according to the 2024 Kyoto International Consensus Criteria [6].

For each participant, separate regions of interest (ROIs) were placed within the head, body and tail of the pancreas. Care was taken to avoid inclusion of major vascular structures, the main pancreatic duct, cystic lesions, and imaging artifacts that could affect measurement accuracy. Three separate measurements were obtained from each pancreatic segment, and the mean value for each segment was calculated. The mean pancreatic fat fraction was subsequently determined as the average of the head, body, and tail measurements.

To ensure measurement consistency and reproducibility, all ROI areas were standardized to a minimum size of 1 cm^2^ whenever anatomically feasible. This methodology allowed objective evaluation of both regional and global pancreatic fat accumulation.

Statistics: The normality of continuous variables was assessed using the Kolmogorov–Smirnov and Shapiro–Wilk tests, together with visual inspection of histograms and Q-Q plots. Continuous variables with normal distribution were presented as mean ± standard deviation, whereas non-normally distributed variables were expressed as median with minimum–maximum values. Categorical variables were presented as numbers and percentages. Comparisons between the IPMN and non-IPMN groups were performed according to variable distribution. Normally distributed continuous variables were compared using the independent samples *t*-test, while non-normally distributed continuous variables were compared using the Mann–Whitney U test. Categorical variables were compared using the chi-square test or Fisher’s exact test, as appropriate. To further evaluate the potential confounding effect of age on pancreatic fat fraction, an analysis of covariance (ANCOVA) was performed using age as a covariate and IPMN status as the fixed factor. Mean pancreatic fat fraction was used as the dependent variable. Adjusted group differences were assessed after controlling for age, and statistical significance was determined using two-sided *p* values. Receiver operating characteristic curve analysis was performed to evaluate the diagnostic performance of pancreatic fat fraction parameters for discriminating IPMN-positive and IPMN-negative patients. The area under the curve, standard error, 95% confidence interval, and *p* values were calculated. Optimal cut-off values were determined using the Youden index, calculated as sensitivity + specificity − 1. The cut-off point with the maximum Youden index was selected as the optimal threshold, and the corresponding sensitivity and specificity values were reported. ROC curve analysis was performed using SPSS Version 26.0 (IBM Corp., Armonk, NY, USA), while Youden index calculations were confirmed using Version 2.6.44 (The jamovi Project, Sydney, NSW, Australia). Univariate logistic regression analysis was performed to identify factors associated with the presence of IPMN. Variables with a *p* value < 0.10 in univariate analysis were entered into multivariable logistic regression models. Because regional and mean pancreatic fat fraction parameters were expected to be highly correlated, each pancreatic fat fraction parameter was evaluated in a separate multivariable logistic regression model. Results were expressed as odds ratios with 95% confidence intervals. A two-tailed *p* value < 0.05 was considered statistically significant. Statistical analyses were performed using IBM SPSS Statistics software (IBM Corp., Armonk, NY, USA).

## 3. Results

In this study, the demographic, anthropometric, and laboratory characteristics of patients with IPMN and control subjects were compared (Table 1). Patients in the IPMN group were significantly older than those in the control group (72.5 [40–93] vs. 57.0 [44–69] years, p = 0.001). Glucose levels were significantly higher in the IPMN group (97.5 [66–259] vs. 95.0 [69–180] mg/dL, p = 0.008), whereas HDL cholesterol levels were significantly lower (40.5 [13–79] vs. 46.0 [30–78] mg/dL, p = 0.008). Hematocrit values were also significantly lower in patients with IPMN compared with controls (40.6 [23.0–50.9]% vs. 42.0 [30.9–50.0]%, *p* = 0.001). In contrast, body mass index, triglyceride, creatinine, ALT, GGT, amylase, and WBC counts did not differ significantly between the two groups (*p* = 0.786, *p* = 0.322, *p* = 0.427, *p* = 0.511, *p* = 0.361, *p* = 0.392, and *p* = 0.293, respectively). To evaluate the potential confounding effect of age, an ANCOVA was performed using age as a covariate. After adjustment for age, mean pancreatic fat fraction remained significantly higher in patients with IPMN than in controls (F = 212.950, *p* < 0.001), whereas age was not independently associated with mean pancreatic fat fraction (F = 0.438, *p* = 0.509) (Supplementary Table S1).

Pancreatic fat fraction measurements in the head and tail regions of the pancreas were significantly higher in patients with IPMN than in control subjects. The median head fat fraction was 14.55 ± 5.85% in the IPMN group compared with 5.30% (0.13–13.10) in the control group (*p* = 0.001). Similarly, the tail fat fraction was significantly increased in the IPMN group (14.95 ± 5.79% vs. 6.95% [3.00–22.10], *p* = 0.001), indicating greater pancreatic fat accumulation in patients with IPMN (Table 2).

In univariate logistic regression analysis, both age and LDH levels were significantly associated with the presence of IPMN. Each one-year increase in age was associated with an 18.0% increase in the odds of IPMN (OR: 1.180, 95% CI: 1.123–1.240, *p* < 0.001), while higher LDH levels were also associated with increased IPMN risk (OR: 1.018, 95% CI: 1.009–1.028, *p* < 0.001). However, after adjustment for potential confounding variables in the multivariable model, age remained an independent predictor of IPMN (adjusted OR: 1.176, 95% CI: 1.071–1.291, *p* = 0.001), whereas the association between LDH and IPMN was no longer statistically significant (adjusted OR: 1.003, 95% CI: 0.978–1.029, *p* = 0.797). HDL cholesterol was not significantly associated with IPMN in the univariate analysis (OR: 0.970, 95% CI: 0.938–1.004, *p* = 0.080) and was therefore not retained in the multivariable model (Table 3).

Multivariable logistic regression analyses demonstrated that age remained an independent predictor of IPMN in all regional pancreatic fat fraction models (all *p* < 0.001). Pancreatic fat fraction was independently associated with IPMN regardless of the pancreatic region evaluated, with the strongest association observed for the pancreatic head (adjusted OR, 1.827; 95% CI, 1.449–2.303; *p* < 0.001), followed by the body (adjusted OR, 1.697; 95% CI, 1.408–2.046; *p* < 0.001) and the tail (adjusted OR, 1.386; 95% CI, 1.213–1.583; *p* < 0.001). Glucose level was independently associated with IPMN only in the tail fat fraction model (adjusted OR, 1.023; 95% CI, 1.001–1.046; *p* = 0.043), whereas LDH was not independently associated with IPMN in any of the models (Table 4).

**Figure 1:**
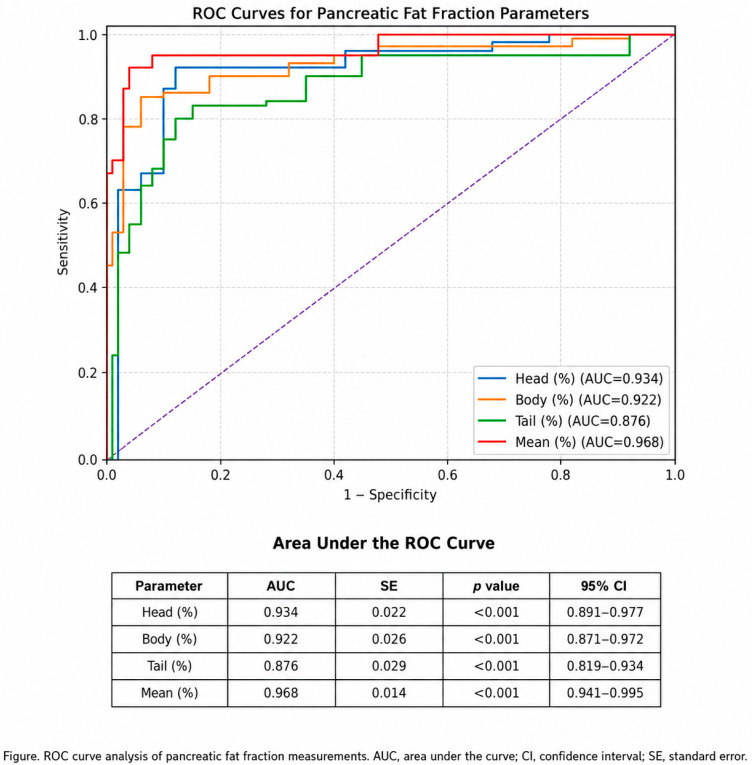
Diagnostic Performance of Pancreatic Fat Fraction Parameters in ROC Analysis.

ROC curve analysis demonstrated that all pancreatic fat fraction parameters had significant discriminative ability for identifying patients with IPMN (all *p* < 0.001). Among the evaluated parameters, the mean pancreatic fat fraction showed the highest diagnostic performance, with an AUC of 0.968 (95% CI: 0.941–0.995), followed by the head fat fraction (AUC = 0.934, 95% CI: 0.891–0.977), body fat fraction (AUC = 0.922, 95% CI: 0.871–0.972), and tail fat fraction (AUC = 0.876, 95% CI: 0.819–0.934). These findings indicate that quantitative MRI-derived pancreatic fat fraction measurements possess excellent diagnostic accuracy for distinguishing patients with IPMN from control subjects, with the mean pancreatic fat fraction demonstrating the greatest discriminative capability (Figure 1).

Receiver operating characteristic curves demonstrate the diagnostic performance of pancreatic fat fraction measurements obtained from the head, body, tail, and mean pancreatic regions. Mean pancreatic fat fraction showed the highest discriminative performance, with an AUC of 0.968, followed by head fat fraction (AUC = 0.934), body fat fraction (AUC = 0.922), and tail fat fraction (AUC = 0.876).

ROC curve analysis identified an optimal head fat fraction cut-off value of 8.45%, which yielded a sensitivity of 91.7% and a specificity of 85.0%, with a Youden index of 0.767 for the detection of IPMN. For the tail fat fraction, the optimal cut-off value was 9.75%, providing a sensitivity of 83.3% and a specificity of 84.2%, with a Youden index of 0.675. These findings indicate that the head fat fraction demonstrated superior diagnostic performance compared with the tail fat fraction for discriminating patients with IPMN from control subjects (Table 5).

## 4. Discussion

In this study, the pancreatic fat fraction measured quantitatively by MRI was found to be significantly and independently associated with the presence of IPMN. Compared with the control group, the IPMN group exhibited significantly higher fat fractions in the head, body and tail regions of the pancreas. Furthermore, multivariable logistic regression analyses demonstrated that age, together with pancreatic fat fraction, was an independent predictor of the presence of IPMN. In ROC analysis, the mean pancreatic fat fraction showed the highest diagnostic performance, with a sensitivity of 91.7% and a specificity of 95.0%, suggesting that pancreatic fat accumulation may represent a promising imaging biomarker for the identification of individuals with IPMN. Several mechanisms may explain the observed association between pancreatic steatosis and IPMN. Excessive fat accumulation within the pancreatic parenchyma promotes chronic low-grade inflammation through the release of pro-inflammatory cytokines and adipokines, induces oxidative stress and fibrosis, and contributes to insulin resistance and hyperinsulinemia, leading to activation of IGF-1–related signaling pathways that may favor neoplastic transformation and epithelial proliferation [7,8,9]. Furthermore, adipose tissue-derived inflammatory mediators may remodel the pancreatic microenvironment and create conditions conducive to tumor initiation and progression [10,11].

In recent years, the relationship between pancreatic steatosis and pancreatic neoplasms has attracted increasing attention. Pancreatic fat accumulation has been shown to represent not only a metabolic condition but also a pathological process associated with chronic inflammation, fibrosis, insulin resistance, and pancreatic carcinogenesis. In particular, MRI-based studies have demonstrated that the amount of pancreatic fat strongly correlates with histopathological fat infiltration and can reflect pancreatic microstructural changes. Therefore, pancreatic steatosis is currently regarded not only as a metabolic finding but also as a potential risk phenotype for the development of pancreatic malignancy [1,2,3].

One of the most important findings of our study was the significantly higher fat fraction observed in all anatomical segments of the pancreas in patients with IPMN. This finding is consistent with the results of current MRI studies investigating the relationship between pancreatic parenchymal characteristics and IPMN risk stratification. Maeba et al. reported that quantitative MRI parameters are associated with IPMN risk stratification and may reflect pancreatic parenchymal alterations as well as lesion biology. Similarly, our study demonstrated a strong association between pancreatic fat infiltration and the presence of IPMN [9].

In regional analyses, the fat fraction in the pancreatic head demonstrated the strongest independent association with IPMN. Multivariable analysis showed that each 1% increase in the pancreatic head fat fraction was associated with an approximately 1.8-fold increase in the likelihood of IPMN. Considering that IPMN lesions are most commonly located in the pancreatic head, this finding is clinically noteworthy. It may be hypothesized that regional fat infiltration alters the local inflammatory microenvironment and facilitates neoplastic transformation [12]. Nevertheless, whether this relationship is causal cannot be determined based on the current study design.

The identification of age as an independent risk factor in all models was an expected finding. Previous epidemiological studies have demonstrated that the prevalence of pancreatic cystic lesions, particularly IPMNs, increases significantly with advancing age [13]. In an abdominal MRI prevalence study, the frequency of pancreatic cysts was shown to increase markedly with age, and approximately half of the detected lesions were reported to be IPMNs. Similarly, recent meta-analyses have shown that the prevalence of IPMN is higher in older populations [14]. Notably, in our study, pancreatic fat fraction remained significant even after adjustment for age. This finding suggests that pancreatic steatosis may not simply be a consequence of aging but may also have an independent association with the presence of IPMN.

Although serum glucose, HDL, and LDH levels differed significantly between the groups in the univariate analysis, these associations disappeared after adjustment for pancreatic fat fraction and age. One possible explanation is that these metabolic abnormalities are systemic manifestations of pancreatic steatosis rather than independent determinants of IPMN. Pancreatic fat accumulation is closely associated with insulin resistance, dyslipidemia, chronic inflammation, and oxidative stress, which may alter circulating glucose and lipid metabolism while simultaneously promoting a pro-neoplastic pancreatic microenvironment. Consequently, pancreatic fat fraction may better reflect the local pathological process than circulating biochemical markers alone, attenuating the independent effects of glucose, HDL, and LDH in multivariable models [8,9,15,16].

The finding that the mean pancreatic fat fraction yielded an AUC value of 0.968 in the ROC analysis is particularly noteworthy. These findings suggest that MRI-derived quantitative pancreatic fat fraction measurements may provide high diagnostic accuracy for identifying patients with IPMN. Current studies indicate that the PDFF method is a reliable and reproducible technique for the assessment of pancreatic steatosis [17]. The optimal cut-off value of 8.81% identified in our study may serve as a clinically useful reference threshold for identifying individuals at increased risk of IPMN in future studies.

The present study has several strengths. First, it provides a relatively comprehensive evaluation of the association between quantitatively measured MRI-derived pancreatic fat fraction and the presence of IPMN. By assessing fat deposition in the head, body, tail, and whole pancreas, the study offers a detailed regional analysis of pancreatic steatosis. Second, the association between pancreatic fat fraction and IPMN remained significant after adjustment for demographic and metabolic variables, supporting the consistency of the observed findings. Third, the excellent diagnostic performance demonstrated in the ROC analysis, particularly for the mean pancreatic fat fraction, suggests that quantitative MRI-based pancreatic fat assessment may have potential utility as a non-invasive imaging marker for identifying individuals at increased risk of IPMN. Finally, the use of quantitative MRI-derived fat fraction measurements provides an objective and reproducible method for evaluating pancreatic steatosis, which may facilitate future investigations into the relationship between ectopic pancreatic fat accumulation and pancreatic cystic neoplasms.

This study has several limitations. First, due to its retrospective and single-center design, selection bias cannot be completely excluded. Second, although the IPMN group had a higher prevalence of diabetes than the control group, suggesting a potential confounding effect, pancreatic fat fraction remained independently associated with IPMN after multivariable adjustment. However, pancreatic fat fraction remained independently associated with IPMN after multivariable adjustment, suggesting that the observed association was not solely explained by differences in diabetes prevalence. Third, histopathological confirmation was not available for all cases. In addition, the inclusion of only normal-weight individuals without metabolic syndrome, smoking history, or excessive alcohol consumption may limit the generalizability of our findings; however, this approach was used to reduce confounding from established metabolic and lifestyle factors associated with pancreatic fat accumulation. Finally, because long-term follow-up data were unavailable, the impact of pancreatic steatosis on malignant transformation or disease progression could not be evaluated.

## 5. Conclusions

MRI-derived pancreatic fat fraction was significantly increased in patients with IPMN and remained independently associated with IPMN presence after adjustment for potential confounding factors. Mean pancreatic fat fraction demonstrated excellent diagnostic performance, suggesting that quantitative assessment of pancreatic steatosis may serve as a valuable imaging biomarker for identifying individuals with IPMN. These findings support a potential link between pancreatic fat accumulation and IPMN and highlight the need for prospective studies to clarify the underlying pathophysiological mechanisms and clinical implications.

## Figures and Tables

**Table 1:** Comparison of Clinical, Metabolic, and Laboratory Parameters Between IPMN and Non-IPMN Groups.

Variables	Non-IPMN (*n* = 120)	IPMN (*n* = 60)	*p*-Value
Age (years)	57.0 (44–69)	72.5 (40–93)	0.001
Gender (F/M)	63/57	31/29	0.521
BMI (kg/m^2^)	22.1 (19.1–24.9)	22.4 (19.0–24.8)	0.786
Waist circumference (cm)	70.47 ± 5.62	70.42 ± 5.07	0.959
Glucose (mg/dL)	95.0 (69–180)	97.5 (66–259)	0.008
Total cholesterol (mg/dL)	198.27 ± 24.84	192.00 ± 48.88	0.353
HDL (mg/dL)	46.0 (30–78)	40.5 (13–79)	0.008
LDL (mg/dL)	121.66 ± 23.45	126.65 ± 41.91	0.394
Triglycerides (mg/dL)	131.5 (54–271)	140.0 (50–439)	0.322
Urea (mg/dL)	38.5 (19–70)	42.0 (14–146)	0.069
Creatinine (mg/dL)	0.96 ± 0.18	0.90 (0.50–2.00)	0.427
AST (U/L)	20.0 (10–71)	19.0 (12–163)	0.888
ALT (U/L)	22.5 (10–81)	19.0 (8–282)	0.511
ALP (U/L)	72.0 (41–108)	69.0 (37–611)	0.589
GGT (U/L)	30.0 (9–86)	22.0 (8–323)	0.361
LDH (U/L)	186.5 (120–244)	193.5 (141–365)	0.002
Amylase (U/L)	82.5 (31–129)	64.0 (20–383)	0.392
Lipase (U/L)	39.0 (20–66)	34.0 (12–135)	0.021
WBC (×10^9^/L)	6.90 ± 1.26	7.19 (4.09–13.49)	0.293
Hemoglobin (g/dL)	13.82 ± 1.30	13.5 (8.0–17.0)	0.020
Hematocrit (%)	42.0 (30.9–50.0)	40.6 (23.0–50.9)	0.001
Platelet count (×10^9^/L)	257.13 ± 48.45	237.75 ± 81.04	0.010

Abbreviations: LDL, low-density lipoprotein; AST, aspartate aminotransferase; ALP, alkaline phosphatase; LDH, lactate dehydrogenase; WBC, white blood cell count; BMI, body mass index.

**Table 2:** Distribution of Pancreatic Fat Fraction in the Head, Body, Tail, and Whole Pancreas According to IPMN Status.

Variables	Non-IPMN (*n* = 120)	IPMN (*n* = 60)	*p* Values
**Head Fat Fraction (%)**	5.30 (0.13–13.10)	14.55 ± 5.85	0.001
**Body Fat Fraction (%)**	6.10 (2.10–15.70)	15.35 ± 5.41	0.001
**Tail Fat Fraction (%)**	6.95 (3.00–22.10)	14.95 ± 5.79	0.001
**Mean Pancreatic Fat Fraction (%)**	4.65 (1.98–12.15)	14.95 ± 5.19	0.001

Abbreviations: IPMN, intraductal papillary mucinous neoplasm. Data are presented as median (minimum–maximum) for non-normally distributed variables and mean ± standard deviation for normally distributed variables.

**Table 3:** Univariate and Multivariable Logistic Regression Analyses of Factors Associated with IPMN Presence: Mean Pancreatic Fat Fraction Model.

Variable	Univariate OR (95% CI)	*p*-Value	Adjusted OR (95% CI)	*p*-Value
**Age (years)**	1.180 (1.123–1.240)	<0.001	1.176 (1.071–1.291)	0.001
**Gender**	0.967 (0.520–1.798)	0.916		
**HDL (mg/dL)**	0.970 (0.938–1.004)	0.080	—	—
**Glucose (mg/dL)**	1.029 (1.014–1.044)	<0.001	1.026 (0.995–1.059)	0.104
**LDH (U/L)**	1.018 (1.009–1.028)	<0.001	1.003 (0.978–1.029)	0.797
**Mean Pancreatic Fat Fraction (%)**	1.081 (1.055–1.108)	<0.001	1.085 (1.051–1.120)	<0.001

Abbreviations: OR, odds ratio; CI, confidence interval; HDL, high-density lipoprotein; LDH, lactate dehydrogenase. Footnote: Variables with *p* < 0.10 in univariate analysis were entered into the multivariable logistic regression model. Age and mean pancreatic fat fraction remained independently associated with the presence of IPMN.

**Table 4:** Logistic Regression Analysis of Factors Associated with IPMN According to Regional Pancreatic Fat Fraction Models.

Variable	Head Model Adjusted OR (95% CI)	*p*	Body Model Adjusted OR (95% CI)	*p*	Tail Model Adjusted OR (95% CI)	*p*
**Age (years)**	1.161 (1.079–1.250)	<0.001	1.164 (1.082–1.252)	<0.001	1.157 (1.086–1.232)	<0.001
**Glucose (mg/dL)**	1.024 (0.999–1.049)	0.059	1.019 (0.995–1.044)	0.113	1.023 (1.001–1.046)	0.043
**LDH (U/L)**	1.003 (0.983–1.023)	0.782	1.009 (0.989–1.029)	0.369	1.011 (0.996–1.026)	0.168
**Pancreatic fat fraction (%)**	Head: 1.827 (1.449–2.303)	<0.001	Body: 1.697 (1.408–2.046)	<0.001	Tail: 1.386 (1.213–1.583)	<0.001

Abbreviations: OR, odds ratio; CI, confidence interval; LDH, lactate dehydrogenase.

**Table 5:** Optimal Cut-off Values of Pancreatic Fat Fraction Parameters for Predicting IPMN Presence.

Parameter	Optimal Cut-off (%)	Sensitivity (%)	Specificity (%)	Youden İndex
**Head fat fraction**	8.45	91.7	85.0	0.767
**Body fat fraction**	9.85	86.7	90.8	0.775
**Tail fat fraction**	9.75	83.3	84.2	0.675
**Mean pancreatic fat fraction**	8.81	91.7	95.0	0.867

## Data Availability

The datasets generated and/or analyzed during the current study are not publicly available due to ethical and privacy restrictions related to patient confidentiality. De-identified data may be made available by the corresponding author upon reasonable request, subject to approval by the relevant institutional ethics committee and applicable data protection regulations.

## Supplementary Materials

The following supporting information can be downloaded at: https://www.mdpi.com/article/10.3390/diagnostics16142198/s1, Table S1. Analysis of Covariance (ANCOVA) for Mean Pancreatic Fat Fraction Adjusted for Age.

## Abbreviations

The following abbreviations are used in this manuscript: The following abbreviations are used in this manuscript: IPMN, intraductal papillary mucinous neoplasm; PDAC, pancreatic ductal adenocarcinoma; PanIN, pancreatic intraepithelial neoplasia; MRI, magnetic resonance imaging; MRCP, magnetic resonance cholangiopancreatography; PDFF, proton density fat fraction; ROI, region of interest; ROC, receiver operating characteristic; AUC, area under the curve; OR, odds ratio; CI, confidence interval; BMI, body mass index; HDL, high-density lipoprotein; LDL, low-density lipoprotein; AST, aspartate aminotransferase; ALT, alanine aminotransferase; ALP, alkaline phosphatase; GGT, gamma-glutamyl transferase; LDH, lactate dehydrogenase; WBC, white blood cell count; SPSS, Statistical Package for the Social Sciences.
