# Supplementary material for: MRI-Derived Pancreatic Fat Fraction Is Independently Associated with Intraductal Papillary Mucinous Neoplasms: A Case–Control Study
Source: Diagnostics (Basel). 2026 Jul 14;16(14):2198. doi: 10.3390/diagnostics16142198 (PMC13408473; doi:10.3390/diagnostics16142198)
Supplement: Supplementary file 1 [file diagnostics-16-02198-s001.zip › diagnostics-4398628-supplementary.pdf]

Supplementary Table S1. Analysis of Covariance (ANCOVA) for Mean Pancreatic Fat Fraction Adjusted for Age

Source	df	F	p-value	Partial η^2
Age (covariate)	1	0.438	0.509	0.002
IPMN status	1	212.950	<0.001	0.546
Error	177	—	—	—
